# Expression of Concern: Escalating spread of SARS-CoV-2 infection after school reopening among students in hotspot districts of Oromia Region in Ethiopia: Longitudinal study

**DOI:** 10.1371/journal.pone.0339229

**Published:** 2025-12-19

**Authors:** The PLOS One Editors

Following the publication of this article [1] and assessment of the underlying data provided to the journal in post-publication discussions, concerns were raised regarding the reference list and sample population. Specifically,

- Concerns were raised about the relevance of articles cited in the Reference list, including Ref. 44 [2].
- A literature search for articles about COVID-19 and schoolchildren published between December 2019 and December 2020 was described in the “Evidence before this study” paragraph in [1], but the described results of this literature search appear to be inaccurate.
- The dataset provided in post-publication discussions includes participants who were older than standard school ages (19–32 years old) despite the study participants being described in [1] as children, and results and conclusions referring to seroprevalence in children.

The corresponding author provided a response regarding the citation of [2]; however, the *PLOS One* Editors do not consider the response to be sufficient to resolve this concern. The author subsequently requested to remove this citation.

The corresponding author provided a list of articles that they claim were the results of the described literature search in the “Evidence before this study” paragraph. These articles, for which no preprints were found, do not appear to meet the search criteria described in [1]. The Editors remain concerned about the validity of claims based on the described literature search but are not concerned that this issue impacts the validity of the study described in this article.

The corresponding author stated that, while the typical age ranges for Ethiopian students are 7–15 years old for primary school and 16–18 years old for secondary school, the inclusion of older participants reflects Ethiopia’s educational context in which accelerated education programs are available to older individuals. The author declined to provide specific details about which schools included in this study offered accelerated education programs. They acknowledged that due to the inclusion of adult participants, the claims regarding children are unsupported by the sample used in this study. The Editors have remaining concerns about the wide age range of the sample population which impacts the interpretation of the results, and about the reproducibility due to lack of information regarding the sources of adult participants. Readers should take this issue into account when interpreting claims in this article related to children.

The *PLOS One* Editors issue this Expression of Concern due to the unresolved issues outlined above.

Contrary to the Data Availability statement in [1], the original raw data files supporting the article’s results were not provided with the article. The authors provided these files following editorial request. They are provided here as S2 and S3 Files.

## Supporting information

S2 File School serological survey results – first round. (XLSX)

S3 File School serological survey results – second round. (XLSX)
