# Supplementary material for: Escalating spread of SARS-CoV-2 infection after school reopening among students in hotspot districts of Oromia Region in Ethiopia: Longitudinal study
Source: PLoS One. 2023 Feb 3;18(2):e0280801. doi: 10.1371/journal.pone.0280801 (PMC9897530; doi:10.1371/journal.pone.0280801)
Supplement: S1 File — (DOCX) [file pone.0280801.s001.docx]

Supporting information 1:Supporting information consiting of all supporting tables and figures

Selected Hot spot Zones and towns with their COVID-19 Confirmed Caseload, as of October 14, 2020 (Regional Surveillance data during site selection and sample size determination)

| Hot spot Zones /Towns | Hotspot Town/Districts | # Of Cases |
| --- | --- | --- |
| Bishoftu Town | Bishoftu Town | 1062 |
| Dukem Town | Dukem Town | 1016 |
| Adama Town | Adama Town | 920 |
| Burayu Town | Burayu Town | 724 |
| Sebata Town | Sebata Town | 541 |
| Assela Town | Assela Town | 512 |
| Nekemte Town | Nekemte Town | 434 |
| Jimma | Seka Chekorsa | 99 |
| East Shewa | Ada'a | 783 |
| West Wollega | Gimbi Town | 248 |
| Arsi | ZiwayDugda | 177 |
| FinfineSorrunding | Sululta Woreda | 139 |
| East Harerge | Babile | 131 |
| West Shewa | Ada'aBarga | 100 |
| Bale | Gasara | 82 |
| Total # of cases from selected Towns and Districts | | 6968 |
| Total # of cases in the Oromia Region as of Oct 15,2020 | | 14,064 |
| Proportion (%) of cases from selected Hot spot Towns and districts | | 49.5 % |

SARS-CoV-2 Sero epidemiology Sampling scheme in selected hotspot towns and districts in the Oromia region, Ethiopia

Change in SARS-CoV-2 seroprevalence during the time of school reopening among selected hotspot areas from December 2020 to April 2021

Availability o**f** Surveillance and Case Management tools in the surveyed schools(N=60), Districts, /towns, Oromiya, Ethiopia

| S/N | Activities | Availability | Frequency (N=60) | Percent |
| --- | --- | --- | --- | --- |
| 1 | Training for staff on COVID_19 prevention and Control | Yes | 47 | 78.3 |
| 2 | Arrangement of communication, transportation and referral system for a suspected case | Yes | 28 | 46.7 |
| 3 | Availability of a temporary isolation room in the school | Yes | 35 | 58.3 |
| 4 | Presence of posted COVID-19 prevention messages | Yes | 33 | 55 |
| 5 | Availability of COVID_19 case definition and hotlines in the compound | Yes | 14 | 23.3 |
| 6 | The school has a Surveillance registration and reporting format | Yes | 12 | 20 |
| 7 | Availability of Communication Channel b/n Schools and Health facilities | Yes | 38 | 63.3 |
| 8 | Availability of COVID_19 multi-sectoral team in the school | Yes | 39 | 65 |
| 9 | Training for students on COVID_19 prevention and Control | Yes | 50 | 83.3 |
| 10 | Classroom desks are at least one meter apart | Yes | 42 | 70 |
| 11 | The school has a tracking mechanism for the absence of students or staff due to COVID_19 | Yes | 43 | 71.7 |

Implementation status of activities related to Policy, guidance, and IPC/ the surveyed schools(N=60), Districts, /towns, Oromiya, Ethiopia

| Activities | **Implementation status** | **Freq(N=60)** | Percent |
| --- | --- | --- | --- |
|  |  |  |  |
| Hand hygiene is adequately exercised in the school by the staff | Always | 20 | 33.3 |
|  | Mostly | 14 | 23.3 |
|  | Not applicable | 1 | 1.7 |
|  | Not at all | 7 | 11.7 |
|  | Sometimes | 18 | 30 |
| COVID-19 prevention awareness for a student is provided regularly | Always | 14 | 23.3 |
|  | Mostly | 13 | 21.7 |
|  | Not applicable | 2 | 3.3 |
|  | Not at all | 5 | 8.3 |
|  | Sometimes | 26 | 43.3 |
| COVID_19 prevention awareness for staff is provided regularly | Always | 14 | 23.3 |
|  | Mostly | 11 | 18.3 |
|  | Not applicable | 2 | 3.3 |
|  | Not at all | 5 | 8.3 |
|  | Sometimes | 28 | 46.7 |
| A physical distancing rule of at least 1 meter between people is implemented | Always | 3 | 5.0 |
|  | Mostly | 13 | 21.7 |
|  | Not applicable | 3 | 5.0 |
|  | Not at all | 16 | 26.7 |
|  | Sometimes | 25 | 41.7 |
| Students and staff do not share or borrow materials or objects from each other | Always | 10 | 16.7 |
|  | Mostly | 9 | 15.0 |
|  | Not applicable | 3 | 5.0 |
|  | Not at all | 15 | 25.0 |
|  | Sometimes | 23 | 38.3 |
| Water is available for hand washing | Always | 35 | 58.3 |
|  | Mostly | 11 | 18.3 |
|  | Not at all | 5 | 8.3 |
|  | Sometimes | 9 | 15.0 |
| Soap is available for hand washing sites | Always | 28 | 46.7 |
|  | Mostly | 11 | 18.3 |
|  | Not at all | 6 | 10.0 |
|  | Sometimes | 15 | 25.0 |

Infection prevention and COVID-19 control practice in the surveyed schools(N=60)

| S/N | Activities | Implementation status | Freq | Percent |
| --- | --- | --- | --- | --- |
| 1 | Frequently touched objects are properly cleaned and disinfected with 0.5% | Always | 7 | 11.7 |
|  |  | Mostly | 5 | 8.3 |
|  |  | Not applicable | 3 | 5 |
|  |  | Not at all | 27 | 45 |
|  |  | Sometimes | 18 | 30 |
| 2 | Availability of functional, sufficient, and well-ventilated toilets with hand washing facilities | Always | 20 | 33.3 |
|  |  | Mostly | 10 | 16.7 |
|  |  | Not applicable | 1 | 1.7 |
|  |  | Not at all | 12 | 20 |
|  |  | Sometimes | 17 | 28.3 |
| 3 | Availability of sufficient no-touch dust bins with lids in all required places | Always | 8 | 13.3 |
|  |  | Mostly | 6 | 10 |
|  |  | Not applicable | 3 | 5 |
|  |  | Not at all | 31 | 51.7 |
|  |  | Sometimes | 12 | 20 |
| 4 | Ways of waste collection handling and disposal methods are safe | Always | 17 | 28.3 |
|  |  | Mostly | 11 | 18.3 |
|  |  | Not at all | 20 | 33.3 |
|  |  | Sometimes | 12 | 20 |
|  |  |  |  |  |
| 5 | Screening for COVID_19 is appropriately practiced in the school | Always | 10 | 16.7 |
|  |  | Mostly | 7 | 11.7 |
|  |  | Not applicable | 5 | 8.3 |
|  |  | Not at all | 29 | 48.3 |
|  |  | Sometimes | 9 | 15 |
| 6 | School practice identification of sick students | Always | 10 | 16.7 |
|  |  | Mostly | 8 | 13.3 |
|  |  | Not applicable | 5 | 8.3 |
|  |  | Not at all | 21 | 35 |
|  |  | Sometimes | 16 | 26.7 |
| 7 | Hand sanitizer is adequately available in the school for students | Always | 10 | 16.7 |
|  |  | Mostly | 10 | 16.7 |
|  |  | Not applicable | 1 | 1.7 |
|  |  | Not at all | 22 | 36.7 |
|  |  | Sometimes | 17 | 28.3 |
| 8 | hand washing facilities and sanitizers are available for staff in the school | Always | 17 | 28.3 |
|  |  | Mostly | 17 | 28.3 |
|  |  | Not at all | 10 | 16.7 |
|  |  | Sometimes | 16 | 26.7 |
| 9 | The cleaning staffs have sufficient cleaning materials and supplies | Always | 16 | 26.7 |
|  |  | Mostly | 9 | 15 |
|  |  | Not applicable | 1 | 1.7 |
|  |  | Not at all | 15 | 25 |
|  |  | Sometimes | 19 | 31.7 |
